# Transiently produced IgGs enable universal SARS-CoV-2 diagnosis and differentiation recent from past infections

**DOI:** 10.1128/spectrum.00044-25

**Published:** 2025-10-27

**Authors:** Chuchu Li, Jingzhi Li, Chen Dong, Qian Zhen, Xiaoyu Li, Lan Yang, Jingjing Cao, Xiaoxiao Kong, Hua Tian, Lu Zhou, Fengcai Zhu, Hongwei Ma, Liguo Zhu

**Affiliations:** 1 Department of Acute Infectious Disease Control and Prevention, Jiangsu Provincial Center for Disease Control and Prevention 12666, Nanjing, China; 2 Division of Nanobiomedicine, Suzhou Institute of Nano-Tech and Nano-Bionics, Chinese Academy of Sciences 85404 , Suzhou, China; 3 Department of Acute Infectious Disease Control and Prevention, Changzhou Center for Disease Control and Prevention 737309 , Changzhou, China; 4 School of Nano-Tech and Nano-Bionics, University of Science and Technology of China 12652, Hefei, China; National Microbiology Laboratory, Winnipeg, Manitoba, Canada

**Keywords:** DIVH, transiently produced IgGs, infection diagnosis, PPHM, SARS-CoV-2 strains

## Abstract

Early diagnosis and differentiating infected from vaccinated animals (DIVA) are the milestone in mitigation, control, and eradication of virus. However, the continuous evolution of new virus strains has hindered the success of many classical techniques, and no DIVA-compatible vaccines are available for severe acute respiratory syndrome coronavirus 2 (SARS-CoV-2). We here develop a universal diagnostic protein-peptide hybrid microarray (PPHM) for different SARS-CoV-2 strains infections and extend the DIVA concept to differentiate recently infected from historically vaccinated/infected hosts (DIVH) in the context of SARS-CoV-2 using in a single sample. First, we analyzed the amino acid sequence identities, which exceeded 96.6%, among the SARS-CoV-2 strains—Delta, Omicron, and New Omicron—and the wild strain. Based on this analysis, we identified a unique diagnostic combination for each of the three SARS-CoV-2 strains using the same peptide probes from the wild strain. Subsequently, we developed a diagnostic combination without regard to strain specificity, PPHM_SARS-CoV-2_, suitable for all three strains, demonstrating a specificity of 99.0% (99/100) and a sensitivity of 90.2% (175/194). Finally, we successfully achieved DIVH and classified 314 individuals to the classifications of uninfected (57/314, 18.2%), newly vaccinated or infected (5/314, 1.6%), recently infected (78/314, 24.8%), and historically vaccinated or infected (174/314, 55.4%) using PPHM_SARS-CoV-2_.

**IMPORTANCE:**

Severe acute respiratory syndrome coronavirus 2 (SARS‐CoV‐2) has caused global health concerns and led to economic losses reaching trillion dollars since 2019. Many diagnostic techniques are showing great success; for instance, nucleic acid amplification showed high specificity and sensitivity in SARS-CoV-2 infections. However, the results of these techniques can be affected by the continuous evolution of SARS-CoV-2 strains, and these techniques were not compatible with differentiating infected from vaccinated animals (DIVA). To circumvent these unfavorable outcomes, a novel strategy is imperative. In this study, we made an exciting discovery: transiently produced IgGs can be used not only for diagnosis and DIVA but also for differentiating recent infections from past infections (approximately 70 days after the occurrence of vaccination or infection). This protein-peptide hybrid microarray technique will have a positive impact on the mitigation and eradication of coronaviruses.

## INTRODUCTION

The COVID-19 pandemic, caused by the severe acute respiratory syndrome coronavirus 2 (SARS-CoV-2), emerged in December 2019 and rapidly became a global threat (1, 2). With the incessant transmission of COVID-19, SARS-CoV-2 has mutated into a variety of variants due to numerous duplications (3). Preventing the mutation and transmission of SARS-CoV-2 seems to be a big issue. It is widely recognized that controlling the spread of infectious diseases involves three fundamental strategies: eliminating the infection sources, cutting off the infection routes, and protecting the susceptible populations (3). Timely isolation of infected individuals has proven effective in curbing the spread of COVID-19. Therefore, developing a technology that accurately and rapidly identifies COVID-19 infections within a short timeframe is of paramount importance. In serological surveys, through this indicator, we can infer the recent infection rate of SARS-CoV-2 in the population, rather than the cumulative positive rate of this virus. Thus, we can timely assess the disease burden caused by this virus infection.

Three diagnostic assays are commonly used for detecting SARS-CoV-2 infections: (i) nucleic acid amplification test (NAAT), (ii) antigen detection, and (iii) antibody detection. These assays detect viral genetic material, viral proteins, and antibodies in serum, respectively (4). Currently, NAAT is considered the gold standard for confirming COVID-19 infections due to its high sensitivity and specificity. However, the accuracy of NAAT results can be influenced by factors in the pre-analytical, analytical, and post-analytical phases, including sampling, nucleic acid extraction, and analysis (5). Antigen-based detection tests are generally the most straightforward diagnostic assays but tend to have lower specificity and sensitivity compared to NAAT (6). The protein-based assays have been developed and serve as a foundation for serological tests to detect coronaviruses in human serum samples (7, 8). However, positive results can indicate either a past or current infection or a vaccination (9, 10), as the COVID-19 vaccines have been widely administered in the population.

The differentiating infected from vaccinated animals (DIVA) concept is crucial in veterinary medicine, as it allows for differentiating infected and vaccinated animals using a single serum sample, which is essential for the complete eradication of a virus. This concept has been successfully applied to various viruses, such as foot-and-mouth disease virus (11), and pest des petite ruminants virus (PPRV) (12). In the classical DIVA approach, vaccines lacking specific proteins are developed, and animal serum samples containing antibodies against these proteins indicate exposure to the natural infections rather than vaccination. However, developing such marker vaccines is both time-consuming and uncertain (11).

Therefore, a novel serological technology for coronavirus should be capable of rapidly, conveniently, and efficiently detecting various strains of SARS-CoV-2 and should incorporate DIVA functionality in a single serum sample. In our previous research, the peptides synthesized according to the amino acid sequences of structural proteins (e.g., the fusion protein of PPRV and capsid protein of porcine circovirus 2 (PCV2)), along with proteins, were used to create a protein-peptide hybrid microarray (PPHM). This platform detects two distinct types of IgG responses: transiently produced IgGs (TPIs), which are anti-peptide antibodies reflecting short-lived immune responses, and persistently produced IgGs (PPIs), which are anti-protein antibodies associated with longer-lasting immunity (10, 13). The PPHM enabled the successful diagnosis of PPRV and PCV2 infections and supported DIVA strategy implementation (9, 10). Meanwhile, this serological assay utilizes a “digital microarray index” (DMI) to diagnose viral infections, addressing issues of non-reproducible interactions (NRIs) and non-specific interactions (NSIs) (14).

In this study, we aim to establish a standardized protocol for the development of a PPHM that possesses diagnostic capabilities, and to extend the DIVA concept to differentiate recently infected from historically vaccinated/infected hosts (DIVH) within the context of SARS-CoV-2. Initially, we analyzed the amino acid sequence identities between the SARS-CoV-2 wild strain and three other strains: Delta, Omicron, and the New Omicron variant. We then used the same probes on Microarray-#1.5 to generate a unique diagnostic combination for these three SARS-CoV-2 strains. Subsequently, we sought to develop a strain-agnostic diagnostic combination, PPHM_SARS-CoV-2_, applicable to all three strains. Finally, we intended to apply the PPHM_SARS-CoV-2_ to screen serum samples from 314 individuals undergoing physical examination to evaluate its DIVH functionality.

## MATERIALS AND METHODS

### Serum samples

At the verification phase, we used 225 serum samples, which contained 27 negative serum samples collected before 2019, 30 serum samples of SARS-CoV-2 vaccinated over 200 days, and a total of 168 positive (i.e., SARS-CoV-2-infected) serum samples (including 50 Delta [B.1.617.2], 85 Omicron [B.1.1.529], and 33 New Omicron [XBB.1.5]). At the validation phase, we used 294 serum samples, which contained 100 negative serum samples collected before 2019, and a total of 194 positive (i.e., SARS-CoV-2-infected) serum samples (including 92 Delta, 75 Omicron, and 27 new Omicron). All the SARS-CoV-2-infected serum samples were evaluated by real-time PCR detection of SARS-CoV-2 infection. The Delta, Omicron, and New Omicron samples used in this study correspond to the WHO-labeled variants B.1.617.2 (Clade 21A), B.1.1.529 (Clade 21K), and XBB.1.5 (Clade 23A), respectively.

For evaluating DIVH functionality, 314 serum samples were collected from persons who were under physical examination in April 2023.

All serum samples were inactivated at 56°C for 30 min and stored at −20°C before testing.

### Peptides and proteins

By analyzing the amino acid sequence of the SARS-CoV-2 wild strain (MN908947), 20-mer peptides with an overlap of 10 aa residues, partially covering four structural proteins (S, N, M, and E) of SARS-CoV-2, were chemically synthesized by GenScript (Jiangsu, China), and ultimately yielded 136 peptides as peptide probes (Table S1). RBD and N proteins (GenScript, Jiangsu, China) of SARS-CoV-2 and set them as protein probes in the experiments. In a previous study, six probes (S39, S95, S37, N15, S21, and N protein) were excluded for they had excessive antigenicity in the discovery phase (15). In this study, we first used 131 peptides (Table S1, black font), and RBD protein.

SARS-CoV-2 Delta (GenBank No. XBA20213), Omicron (GenBank No. WZA33875), and New Omicron (GenBank No. XBA90971) strains were analyzed using NCBI.

### Microarrays

Microarray-#1.5 was formed with one protein (RBD protein), and 131 peptides (Table S1, black font). Briefly, 0.1 mg/mL of each peptide or protein was printed onto the iPDMS substrate membrane using the non-contact printer sciFLEXARRAYER S1 (Scienion, Berlin, Germany) to form a 12 × 12 array (one microarray with 12 arrays for serum screening). In each array, eight positive controls were printed with human IgG at a concentration of 10 µg/mL, and one negative control was printed with buffer.

PPHM_SARS-CoV-2_ was formed after the verification stage and used for the validation stage of serum screening, which consisted of one RBD protein and eight peptides (including N10, N24, N37, N39, S46, S58, S63, and S71). Briefly, 0.1 mg/mL of each peptide or protein was printed onto the iPDMS substrate membrane using the non-contact printer sciFLEXARRAYER S1 (Scienion, Berlin, Germany) to form a 4 × 4 array (one microarray with 48 arrays for serum screening). In each array, three positive controls were printed with human IgG at a concentration of 10 µg/mL, and one negative control was printed with buffer.

### Serum screening with microarrays

Two hundred twenty-five serum samples used in the verification stage were screened using Microarray-#1.5. Serum was first diluted 1:100 with serum-dilution buffer (1% bovine serum albumin, 1% casein, 0.5% sucrose, 0.2% polyvinylpyrrolidone, and 0.5% Tween20 in 0.01 M phosphate-buffered saline, pH = 7.4) and 450 mL was added into each array of Microarray-#1.5, incubated for 30 min on a shaker (500 rpm, 37°C). One array incubated with serum-dilution buffer was used as a negative control. The microarray was then rinsed three times with washing buffer and incubated with 450 mL/array of horseradish peroxidase (HRP)-conjugated goat anti-human IgG (Sigma-Aldrich) diluted 1:10,000 in Peroxidase Conjugate Stabilizer/Diluent (Thermo Scientific) for another 30 min on a shaker (500 rpm, 37°C), followed by the same washing steps as described above. Then, 100 mL of chemiluminescence substrate (Thermo Scientific) was added onto each array of the microarray, and the images were taken at a wavelength of 635 nm using Clear 4 imaging system (Suzhou Epitope, Suzhou, China). The signal of any dot was defined as signal readout of dot minus signal readout of background.

Two hundred ninety-four serum samples used in the validation stage and 314 serum samples from physical examination were screened using PPHM_SARS-CoV-2_. Serum was first diluted 1:100 with serum-dilution buffer (1% bovine serum albumin, 1% casein, 0.5% sucrose, 0.2% polyvinylpyrrolidone, and 0.5% Tween20 in 0.01 M phosphate-buffered saline, pH = 7.4) and 100 mL was added into each array PPHM_SARS-CoV-2_, incubated for 30 min on a shaker (500 rpm, 37°C). One array incubated with serum-dilution buffer was used as a negative control. The PPHM was then rinsed three times with washing buffer and incubated with 100 mL/array of HRP-conjugated goat anti-human IgG (Sigma-Aldrich) diluted 1:10,000 in Peroxidase Conjugate Stabilizer/Diluent (Thermo Scientific) for another 30 min on a shaker (500 rpm, 37°C), followed by the same washing steps as described above. Then, 90 mL of TMB (Thermo Scientific) was added into each array of the PPHM, incubated at 37°C for 10 min, then rinsed three times with pure water. Finally, the informative signal of IgGs against probes using a microarray imager (Suzhou Epitope). The data were processed using IBT software, which was also developed by Suzhou Epitope. The signal for each dot was calculated using the following equation: signal dot = signal readout − signal background.

### Statistical analysis

We use the formula signal = (peptide signal intensity − background intensity)/(background intensity) to convert the intensity to a signal value and define signal = 2 as the filter value of a single probe. The whole-microarray-level cutoff value (i.e., 2) used a DMI, which represented the overall responding peptide probes on PPHM. ROC was performed using GraphPad Prism. *P* value was calculated by SPSS.

Based on previous studies (10, 13), we defined: both DMI positive and anti-protein positive as recent infection; DMI negative and anti-protein positive as past infection; both DMI and anti-protein negative as uninfected; DMI positive and anti-protein negative as early infection or early seroconversion.

## RESULTS

### Study design

The antibody response is largely contingent upon the ability of antibodies elicited during a natural infection or vaccination to recognize viral antigens upon exposure to the virus. These antibodies may be circulating in the blood, or generated by memory B cells and plasma cells upon re-exposure to the viral antigens (16). In the context of coronaviruses, the majority of antibodies target the SARS-CoV-2 viral antigens, notably the spike surface glycoprotein (S) and the nucleocapsid phosphoprotein (N) (17, 18). The S protein is targeted due to its critical role in viral entry (19), and the N protein is targeted due to its high expression levels during infection (20), rendering both key antigens for antibody recognition.

**Fig 1:**
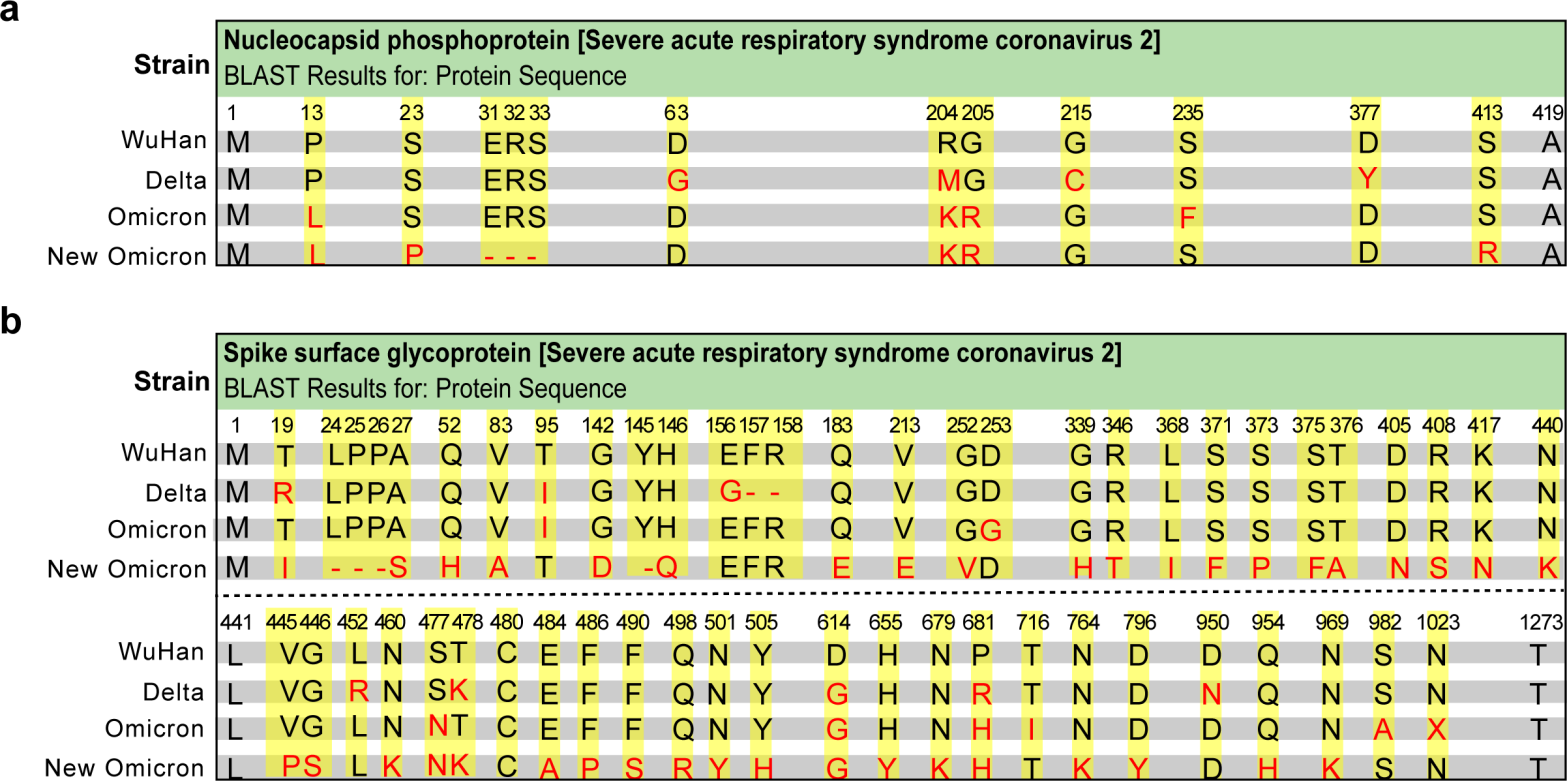
The protein sequence BLAST results between each three SARS-CoV-2 strains and the SARS-CoV-2 wild strain. (**a and b**) The alignments of nucleocapsid phosphoprotein sequence (**a**), spike surface glycoprotein sequence (**b**) showed high identities. Nucleocapsid phosphoprotein sequence had a total of 419 amino acids, and spike surface glycoprotein sequence had a total of 1273 amino acids. The numbers above amino acids indicate the corresponding amino acid locations, the amino acids in red indicate mutations, and the “-” indicates a gap. The SARS-CoV-2 amino acid sequences were analyzed using the data from NCBI GenBank No. MN908947 (wild strain), GenBank No. XBA20213 (Delta strain), GenBank No. WZA33875 (Omicron strain), and GenBank No. XBA90971 (New Omicron strain).

Minor differences in amino acid sequences can significantly influence pathogenicity and variations in antibodies induced by conformational epitope, while differences at the linear epitope level are likely minimal and predictable (21). Consequently, we posit that serological diagnostics based on peptides (i.e., linear epitopes) may effectively cover different strains. If substantial differences in linear epitopes exist or if they elicit distinct humoral immune responses, strain-specific identification may also be feasible. Under this hypothesis, we compared the amino acid sequences of the S and N proteins from four SARS-CoV-2 strains, including wild-type (Wuhan-Hu-1, Clade 19A), Delta (B.1.617.2, Clade 21A), Omicron (B.1.1.529, Clade 21K), and New Omicron (XBB.1.5, Clade 23A). These classifications were based on PANGO lineage and GISAID clade definitions. Despite variant divergence, the amino acid sequence identities of the S and N proteins remained above 96.6% (Fig. 1; Table 1), allowing us to use wild-type-derived peptide probes for diagnostic combination design across variants.

**TABLE 1:** Comparisons of protein sequence identities between each three SARS-CoV-2 strains and SARS-CoV-2 wild strain

Protein	SARS-CoV-2 strains	Identities	Gaps
Nucleocapsid phosphoprotein	Delta	415/419 (99.0%)	0/419 (0%)
	Omicron	415/419 (99.0%)	0/419 (0%)
	New Omicron	411/419 (98.1%)	3/419 (7.2%)
Spike surface glycoprotein	Delta	1,264/1,273 (99.3%)	2/1,273 (0.1%)
	Omicron	1,265/1,273 (99.4%)	0/1,273 (0%)
	New Omicron	1,230/1,273 (96.6%)	4/1,273 (0.3%)

### One unique diagnostic combination for each of the three SARS-CoV-2 strains

As previously reported, a diagnosis combination of 131 peptides was identified during the discovery phase (15). Here, we presented details from the verification phase. Specifically, we used 27 serum samples collected in 2019 (Control 1: negative, pre-COVID-19 outbreak), 30 serum samples from individuals vaccinated over 200 days ago, but confirmed uninfected with SARS-CoV-2 (Control 2), and 50 serum samples from Delta-infected individuals for serological screening using Microarray-#1.5 (containing 132 probes, i.e., 131 peptides and a RBD protein). Ultimately, we identified a diagnostic combination of six peptides for confirming Delta strain infections: S46, S58, S63, S71, S82, and N24 (Combination-Delta).

**Fig 2:**
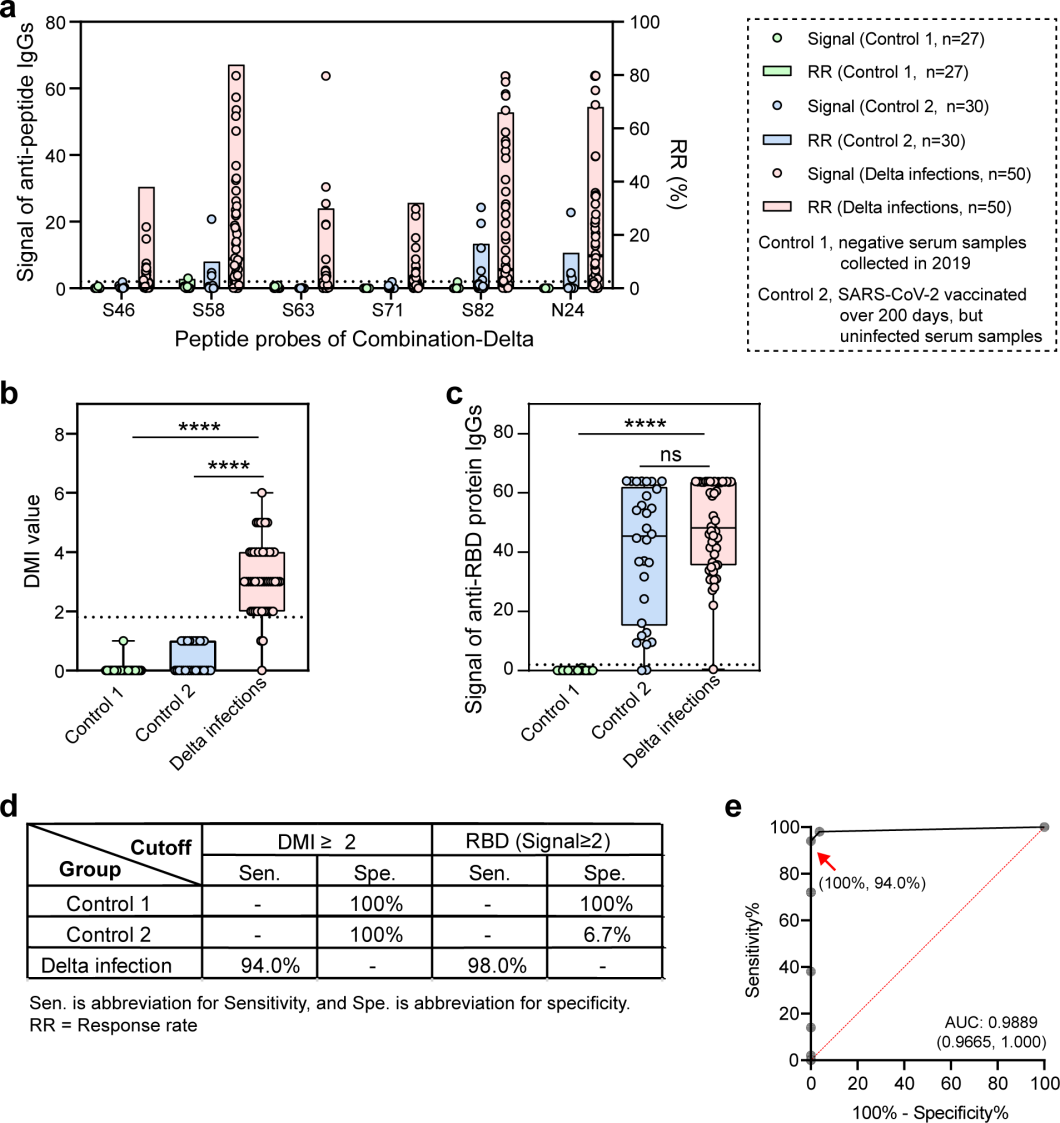
The Combination-Delta effectively differentiates between serum samples from individuals infected with the Delta strain and those from uninfected or historically vaccinated individuals. (**a**) The response signals and rates of the peptide probes (S46, S58, S63, S71, S82, and N24) (i.e., TPIs) in Combination-Delta were evaluated using serum samples from Control 1 (uninfected, pre-COVID-19), Control 2 (vaccinated and uninfected), and Delta-infected groups. (**b**) The DMI values, representing the number of responsive peptide probes, were analyzed across the same groups. Significant differences in DMI values were observed between Control 1 and Delta-infected serum samples (*P* < 0.001), as well as between Control 2 and Delta-infected groups. (**c**) The response signals of the RBD protein (i.e., PPIs) were evaluated in the same groups. Significant differences in RBD response signals were observed between Control 1 and Delta-infected groups (*P* < 0.001), with no significant differences between Control 2 and Delta-infected groups. (**d**) When applying a DMI cutoff of ≥2 for anti-peptide antibodies or a signal value ≥2 for anti-protein antibodies, the groups displayed varying specificities and sensitivities. At a DMI cutoff of ≥2, both control groups showed 100% specificity, with a 94.0% sensitivity for the Delta-infected group. Using RBD protein as a probe with a signal value ≥2, the two control groups had specificities of 100% and 6.7%, respectively, and the Delta-infected group had a sensitivity of 98.0%. (**e**) Using a DMI cutoff ≥2, the Combination-Delta demonstrated a specificity of 100% and a sensitivity of 94.0% for Control 1 and Delta-infected samples, respectively. Statistical significance: ns, not significant; ****P < 0.005.

For Control 1, the serum samples were theoretically negative as they were collected prior to the COVID-19 outbreak, and the results showed no response to SARS-CoV-2 probes (Fig. 2a and c-green). Regarding Control 2, as the samples were collected over 200 days post-vaccination from individuals confirmed uninfected, they were expected to be negative for anti-peptide antibodies (10, 13). The results showed only three peptides responded (peptides S58, S82, and N24), and the positive rates were all below 20% (Fig. 2a). Using a cutoff of DMI ≥ 2, the positive rate for Control 2 was 0% (Fig. 2b). In contrast, the positive rates for the peptides screened with Delta-infected serum samples ranged from 30.0% to 84.0%, exhibiting over a 30% difference in response rate between control and Delta-infected groups (Fig. 2a). This Combination-Delta showed significant differences in responses between controls and Delta-infected groups (*P* < 0.005), achieving 100% specificities and 94.0% sensitivity (Fig. 2b and d), with an area under the ROC curve (AUC) of 0.9889 (Fig. 2e), indicating excellent diagnostic performance.

Protein-based assays utilizing the RBD as a detection antigen can also be developed on Microarray with a signal value cutoff ≥2. Serum samples from Control 2 tested positive for anti-RBD antibodies due to vaccination, indicating that protein-based assays cannot be used for DIVA. Serological screening revealed sensitivity for RBD protein in Delta-infected to be 98.0%, and specificities for Control 1 and Control 2 to be 100% and 6.7%, respectively (Fig. 2d). The responses of anti-RBD were with no significant differences between Control 2 and Delta-infected groups, and with significant differences between Control 1 and Delta-infected groups (*P* < 0.005) (Fig. 2c).

Employing the same methodology used for the Combination-Delta, we applied this approach during the verification phase for diagnosing the Omicron and New Omicron strains. In addition to the two Control groups, we collected 85 positive serum samples from Omicron-infected individuals and 33 from New Omicron-infected individuals to screen using Microarray-#1.5. We then identified a diagnostic combination of seven peptides for the Omicron strain: S46, S58, N10, N14, N16, N37, and N39 (Combination-Omicron) (Fig. S1a). Combination-Omicron showed significant differences in responses between controls and Omicron-infected groups (*P* < 0.005), achieving 100% (Control 1) and 93.3% (Control 2) specificities (Fig. S1b and d), and 92.9% sensitivity (Fig. S1b and d), with an AUC of 0.9634 (Fig. S1e). Furthermore, we developed a diagnostic combination for the New Omicron strain, comprising five peptides and an RBD protein: S46, S71, N25, N37, N39, and RBD (Combination-New Omicron) (Fig. S2a). The inclusion of the RBD protein may be necessary due to the limited recognition between the peptide and antibodies induced by infections with the new Omicron variant. Combination-New Omicron showed significant differences in responses between controls and New Omicron-infected groups (*P* < 0.005), achieving 100% (Control 1) and 96.7% (Control 2) specificities (Fig. S2b and d), and 90.9% sensitivity (Fig. S2b and d), with an AUC of 0.9983 (Fig. S2e).

The results of these three diagnostic combinations suggest that the DMI can not only be used for infectious diagnosis, but also be able to DIVA.

### A diagnostic combination suitable for all three SARS-CoV-2 strains

**Fig 3:**
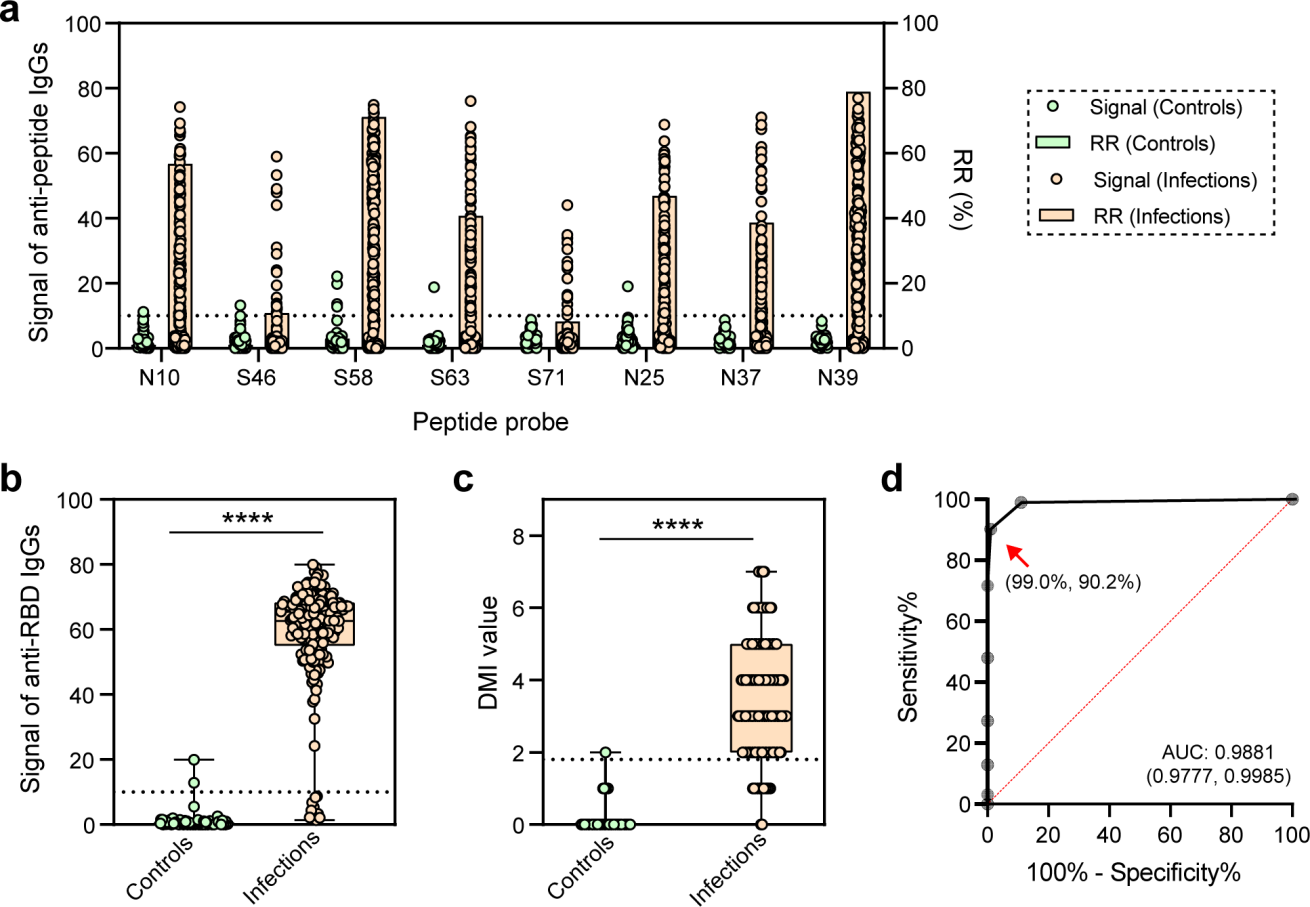
The PPHM_SARS-CoV-2_ demonstrated a diagnostic sensitivity of 90.2% for all SARS-CoV-2-infected serum samples. (**a**) The response signals and rates of the peptide probes (N10, S46, S58, S63, S71, N25, N37, and N39) (i.e., TPIs) when screened against negative (*n* = 100) and positive (*n* = 194) serum samples were analyzed. (**b**) The response signals of the RBD protein (i.e., PPIs) when screened against negative and positive serum samples indicated a significant difference (*P* < 0.001). RBD demonstrated a specificity of 98.0% and a sensitivity of 94.3% for negative and positive serum samples, respectively. (**c**) The DMI values revealed a significant difference in positive and negative serum samples (*P* < 0.001). (**d**) Using a DMI cutoff ≥2, the PPHM_SARS-CoV-2_ demonstrated a specificity of 99.0% and a sensitivity of 90.2% for negative and positive serum samples, respectively. Statistical significance: ****P < 0.005.

As previously mentioned, serum samples infected with three SARS-CoV-2 strains were initially screened using the same set of probes (i.e., Microarray-#1.5), with amino acid sequence identities reaching 96.6% between the peptide probes from the wild strain and each of the three SARS-CoV-2 strains (Fig. 1). The screening results further indicated cross-reactivity among serum samples infected with different SARS-CoV-2 strains when using the unique diagnostic combination for each strain (Table 2): (i) Combination-Delta exhibited diagnostic sensitivities of 67.1% and 81.8% for Omicron and New Omicron strains, respectively; (ii) Combination-Omicron showed sensitivities of 86.0% and 75.8% for Delta and New Omicron strains, respectively; and (iii) Combination-New Omicron displayed sensitivities of 86.0% and 80.0% for Delta and Omicron strains, respectively. These data suggested that cross-reactivity presented a challenge in achieving strain-specific diagnostic combination. To facilitate screening, we developed a new combination (N10, N24, N37, N39, S46, S58, S63, and S71), along with RBD protein, named PPHM_SARS-CoV-2_, intended for positive diagnosis of SARS-CoV-2 without regard to strain specificity in the verification phase (Fig. 3a).

**TABLE 2:** Cross-reactivities between different SARS-CoV-2 strains

Diagnostic combination	Serum samples^*a*^		
	Delta-infected	Omicron-infected	New Omicron-infected
Combination-Delta (S46, S58, S63, S71, S82, and N24)	(94.0%)	67.1%	81.8%
Combination-Omicron (S46, S58, N10, N14, N16, N37, and N39)	86.0%	(92.9%)	75.8%
Combination-New Omicron (S46, S71, N25, N37, N39, and RBD)	86.0%	80.0%	(90.9%)

^*a*^ Cutoff: DMI ≥ 2. The values in parentheses in represent the sensitivity of each combination against the corresponding target infection.

To validate the feasibility of the PPHM_SARS-CoV-2_, we used 100 negative serum samples collected before 2019, and a positive group comprising 92 Delta-infected, 75 Omicron-infected, and 27 New Omicron-infected serum samples, for serological screening with PPHM_SARS-CoV-2_ in the validation phase. The screening results showed that the positive rates of probes in the 194 positive serum samples ranged from 8.2% to 78.9% (Fig. 3a), with the RBD protein achieving a 94.3% positive rate (Fig. 3b). Ultimately, the PPHM_SARS-CoV-2_ assay using DMI demonstrated a 90.2% sensitivity for positive serum samples and a 99.0% specificity for negative serum samples (Fig. 3c and d), which showed DMI can improve simultaneously sensitivity and specificity beyond what is achievable with a single peptide probe (Fig. S3).

Additionally, we evaluated the sensitivity of the PPHM_SARS-CoV-2_ assay for each SARS-CoV-2 strain, finding sensitivities of 89.1%, 93.3%, and 85.2% in the Delta-infected, Omicron-infected, and New Omicron-infected groups, respectively (Fig. S4). This suggested that the diagnostic performance of the PPHM_SARS-CoV-2_ assay is consistent across different strains. For further differentiation of infecting strains, we could employ NAAT and whole-genome sequencing to identify the specific strain.

### PPHM_SARS-CoV-2_ can DIVH

**Fig 4:**
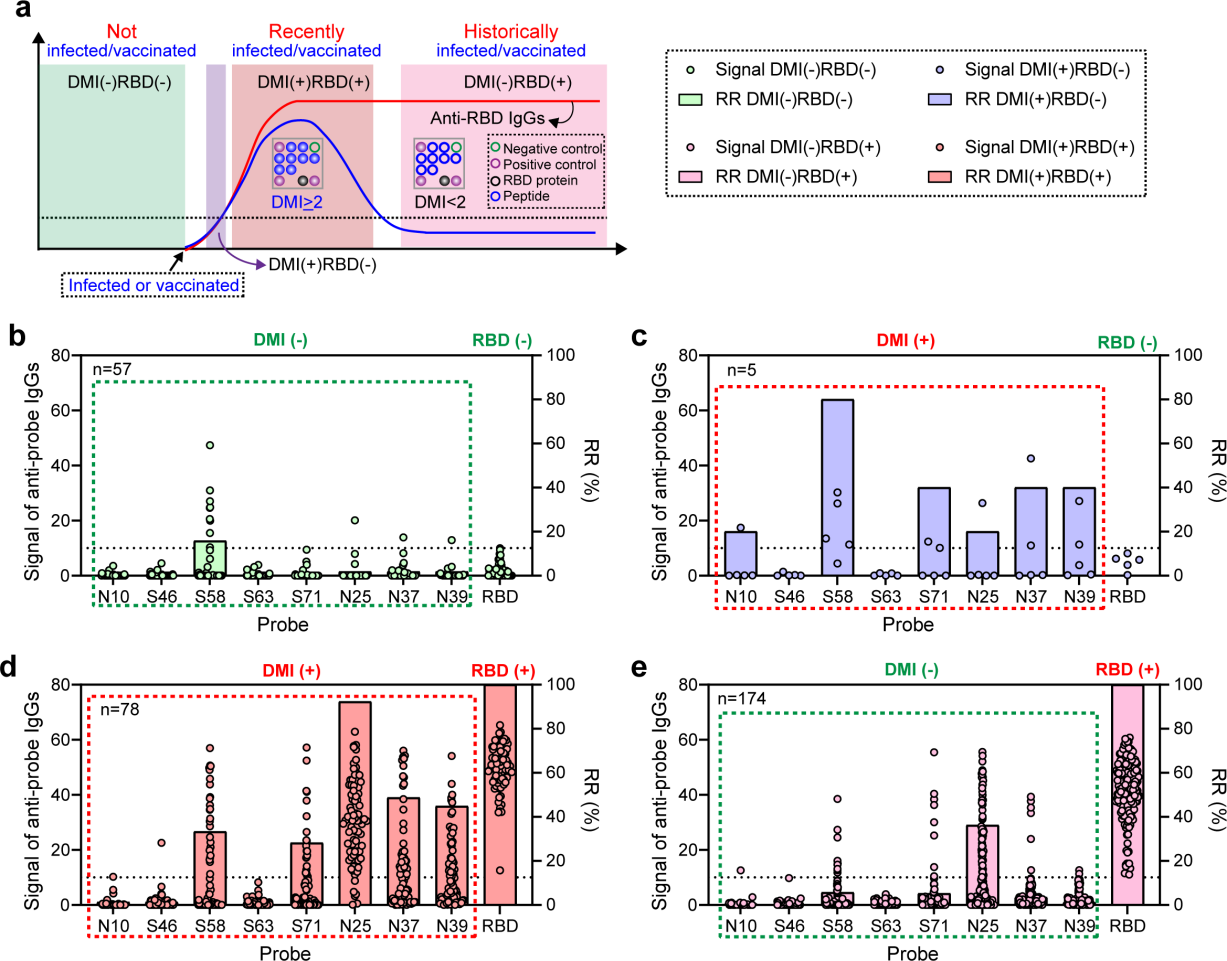
The simultaneous testing of anti-peptide and anti-RBD protein antibodies enables the determination of vaccination or infection status from a single serum sample. (**a**) A schematic diagram for differentiating between past vaccination or infection and recent infection. DMI (−) and RBD (−) indicated unvaccinated and uninfected; DMI (+) RBD (−) indicated the beginning vaccination or infection, where anti-peptide antibodies occurred earlier than anti-protein antibodies. DMI (+) and RBD (+) indicated recent vaccination or infection; and DMI (−) and RBD (+) indicated past vaccination or infection. (**b–e**) Response signals and rates of peptides (N10, S46, S58, S63, S71, N25, N37, and N39) and RBD proteins across various serum classifications are as follows: (**b**) the DMI (−) and RBD (−) classification includes a total of 57 serum samples; (**c**) the DMI (+) and RBD (−) classification includes a total of five serum samples; (**d**) the DMI (+) and RBD (+) classification includes a total of 78 serum samples; (**e**) the DMI (−) and RBD (+) classification includes a total of 174 serum samples.

The ability to achieve DIVH is crucial for using the PPHM to determine recent infections and serves as the foundation for subsequent strain differentiation using NAAT. Previous studies have shown that anti-peptide antibodies are TPIs with a duration of approximately 70 days after the occurrence of vaccination or infection, while anti-protein antibodies are PPIs (10, 13). Therefore, by considering the timing of sampling, and the status of anti-peptide and anti-protein antibodies in a single serum sample, we can further determine whether the serum host has a past vaccination/infection (the occurrence of vaccination or infection events exceeding 70 days) or a recent infection (Fig. 4a). Note that in the screening of PPHM_SARS-CoV-2_, RBD can also be used as one of the detection antigens, which allowed for the detection of both anti-peptide and anti-protein antibodies in a single serum sample and provided the foundation of DIVH. Next, we screened 314 serum samples collected during physical examinations in April 2023 to determine the DIVH ability of PPHM_SARS-CoV-2_.

The screening results identified four classifications (Table 3): (i) DMI (− [negative]) RBD (−), indicating the absence of both anti-peptide and anti-protein antibodies, suggested that these individuals were unvaccinated and uninfected, comprising a total of 57 samples (18.2%) (Fig. 4b); (ii) DMI (+, positive) RBD (−), signifying the presence of anti-peptide antibodies and the absence of anti-protein antibodies; theoretically, this classification may only exist in cases of occurrence of vaccination/infection, where anti-peptide antibodies appeared earlier than anti-protein antibodies, with a total of only five samples (1.6%) (Fig. 4c); (iii) DMI (+) RBD (+), indicating the presence of both anti-peptide and anti-protein antibodies, suggested recent vaccination/infection, with a total of 78 samples (24.8%) (Fig. 4d); and (iv) DMI (−) RBD (+), where anti-peptide antibodies are negative and anti-protein antibodies are positive, indicated past vaccination/infection, as the anti-peptide antibodies induced by past vaccination/infection have disappeared by the time of sampling, with a total of 174 samples (55.4%) (Fig. 4e).

**TABLE 3:** Classification of DMI and anti-RBD protein antibodies in a single serum sample

Classifications		Number	Meaning
DMI	RBD		
Negative (DMI < 2)	Negative	57 (18.2%)	Not vaccinated or infected
	Positive	174 (55.4%)	Historically vaccinated or infected
	Total	231	
Positive (DMI ≥2)	Negative	5 (1.6%)	Maybe newly vaccinated or infected
	Positive	78 (24.8%)	Recently vaccinated or infected
	Total	83	
**Total**		314	

The data indicated that employing both anti-peptide antibodies (DMI) and anti-RBD protein antibodies in a single serum can reveal the vaccination or infection status. Among the serum samples collected in April 2023, over half (55.4%, 174 samples) demonstrated past infections [DMI (−), RBD (+)], and 24.8% indicated recent infections [DMI (+), RBD (+)], which corresponded with China’s situation following the easing of COVID-19 restrictions at the end of December 2022. Furthermore, 18.2% of the population was unvaccinated and uninfected [DMI (−), RBD (−)], with this group predominantly comprising elderly individuals, aligning with the demographic profile in China.

If only anti-peptide antibodies are assessed, we can only determine whether the DMI value is negative or positive. According to the previous research, a negative DMI does not necessarily indicate that the serum sample is from an uninfected or unvaccinated individual, as anti-peptide antibodies are short-lived and disappear over time (13). If only anti-RBD protein antibodies are assessed, it is not possible to DIVH (10). Therefore, simultaneous testing of anti-peptide and anti-RBD protein antibodies is beneficial for accurately determining the vaccination or infection status of a serum sample and is advantageous for epidemiological investigations of COVID-19.

## DISCUSSION

In this study, we demonstrated that PPHM_SARS-CoV-2_ can effectively (i) diagnose infections of SARS-CoV-2 and (ii) achieve DIVH, that is, differentiate recently infected from historically vaccinated/infected hosts. Despite the existence of various diagnostic methods for COVID-19, including NAAT and antigen detection, their effectiveness has often been suboptimal, especially concerning DIVH. Our findings suggested that serological antibody screening with PPHM_SARS-CoV-2_ plays a pivotal role in diagnosing and monitoring COVID-19, thereby contributing to the containment of the virus’s spread.

During the COVID-19 pandemic, the incomplete understanding of antibodies generated by SARS-CoV-2 infections, coupled with prevalent issues such as NSI and NRI in traditional serological assays, has impeded the broad application of serological antibody testing for infection monitoring. Factors like heterophile antibodies (22) and high antibody concentrations (14, 23) can lead to binding with different target antigens, exacerbating NSI/NRI issues. Our PPHM approach recognizes and mitigates these influences by employing a DMI method to counteract their adverse effects.

Conceptually, proteins are regarded as compositions of multiple linear epitopes besides conformational epitopes, offering greater sensitivity than single peptides when utilized as probes in diagnostic assays. For instance, when diagnosing infections with PPHM_SARS-CoV-2_, the sensitivity of the RBD protein was 94.3% (Fig. 3b), whereas each corresponding peptide (S46, S58, S63, and S71) exhibited significantly lower sensitivities: 10.8%, 71.1%, 40.7%, and 8.2%, respectively (Fig. 3a). After applying the DMI analysis, peptides derived from the RBD protein and N protein achieved a sensitivity of 90.2% and specificity of 99.0% for diagnosing SARS-CoV-2 infections (Fig. 3d), indicating that both sensitivity and specificity were improved simultaneously, surpassing the level achievable with a single peptide probe (Fig. S3). The observed higher sensitivity of the RBD protein may be attributed to the implementation of the extensive COVID-19 vaccination campaign in China in 2021, leading to a robust positive signal across populations.

Recall that proteins, being mixtures of linear epitopes, not only possess higher sensitivity but also facilitate the aggregation of signals (24). Recent assays for rapid COVID-19 detection, such as label-free fiber optic surface plasmon resonance (SPR)-based biosensors (25), localized SPR-based nano-plasmonic biosensors (26), and microfluidic-based point-of-care biosensors (27), still face challenges when using proteins as probes, which can lead to aggregated signals. Our previous research highlighted the existence of different longevity antibodies, namely short-lived anti-peptide antibodies termed TPIs, and long-lived anti-protein antibodies termed PPIs, allowing us to develop a DIVH strategy: monitoring whether TPI reappears due to infection after disappearance, without the need of developing marker vaccines (with negative tags) (10, 11). In this study, we verified the presence of both TPIs and PPIs, as well as the DIVH functionality of TPIs. In the detection of serum from individuals uninfected but vaccinated for more than 200 days, the anti-peptide antibodies showed a 100% negative result, indicating that (i) the anti-peptide antibodies were indeed TPIs and (ii) the negative results of anti-peptide antibodies indicated no recent infection. The RBD protein showed a positive rate of 93.3%, also indicating that (i) the anti-protein antibodies were mainly PPIs and can remain positive more than 200 days after vaccination, and (ii) the anti-protein antibodies remained consistently positive and therefore cannot indicate recent infection (Fig. 2; Fig. S1 and S2). In addition, we further categorized an individual’s immune status according to the occurrence and development of anti-peptide and anti-protein antibodies: (i) when DMI (−) RBD (−) occurs, it indicates no vaccination or infection; (ii) when DMI (+) RBD (+) occurs, it indicates recent vaccination or infection; (iii) when DMI (−) RBD (+) occurs, it indicates past vaccination or infection. This criterion provides foundations for determining an individual’s vaccination or infection status solely using a single serum sample.

In an ideal scenario, the detection of anti-protein antibodies should correlate with viral infection, ensuring positive responses to pathogenic protein probes. To minimize NSI/NRI due to molecular mimicry, it is essential to use corresponding pathogenic proteins and their derived peptides as probes. However, RNA viruses like SARS-CoV-2, known for their error-prone replication process, are prone to mutation, thus fostering virus evolution through replication-associated changes (28). This implies that if diagnosis is required for every SARS-CoV-2 infection strain, at least six different detection kits would need to be developed. To alleviate the corresponding workload, we have developed a versatile PPHM_SARS-CoV-2_ assay, capable of diagnosing infections across different strains, based on (i) the high amino acid sequence similarity (up to 96.6%) among strains, (ii) the utilization of linear peptides as probes, and (iii) the ability of DMI to overcome NSI/NRI. The PPHM_SARS-CoV-2_ assay has demonstrated a sensitivity of 90.2% for diagnosing SARS-CoV-2 infections.

Thus, PPHM_SARS-CoV-2_ holds distinct advantages in diagnosing SARS-CoV-2, particularly for differentiating between past vaccination or infection and recent infection. While this study did not include head-to-head comparisons with commercial ELISA or lateral flow assays, we have discussed their conceptual differences in assay design and diagnostic capability. Future benchmarking studies are warranted. However, this study also has several limitations. First, this assay cannot directly identify the infecting strain, and confirmation of the strain type still requires NAAT after infection diagnosis. Second, due to the challenge of obtaining individual continuous serum samples, we were only able to derive estimated occurrence and development curves for anti-protein and anti-peptide antibodies. Moreover, we acknowledge that longitudinal monitoring of antibody dynamics would provide deeper insights into TPIs. Third, potential cross-reactivity with antibodies against other human coronaviruses was not fully evaluated and warrants further investigation. Finally, the relatively limited sample size and lack of validation in diverse populations, such as elderly or immunocompromised individuals, may affect the generalizability of our findings.

With the development of new vaccines to prevent human diseases and the implementation of public health measures, there is a growing demand for the assessment of vaccine efficacy, disease diagnosis, and prognosis. It is essential to develop rapid and reliable assays to meet these assessment needs, such as those for Human Papillomavirus in cervical cancer (29), and Epstein-Barr Virus in nasopharyngeal cancer (30). Our research on diagnosing the SARS-CoV-2 infections has positive implications for the early prevention and diagnosis of cancers caused by these viruses. Moreover, the assays developed for differentiating past vaccination or infection from recent infection present a promising approach for controlling and potentially eradicating the virus.

## SUPPLEMENTAL MATERIAL

Supplemental figures spectrum.00044-25-s0001.docx Figure S1 to S4.

Table S1 spectrum.00044-25-s0002.xlsx Peptide sequence.

ASM does not own the copyrights to Supplemental Material that may be linked to, or accessed through, an article. The authors have granted ASM a non-exclusive, world-wide license to publish the Supplemental Material files. Please contact the corresponding author directly for reuse.
