# Supplementary material for: Transiently produced IgGs enable universal SARS-CoV-2 diagnosis and differentiation recent from past infections
Source: Microbiol Spectr. 2025 Oct 27;13(12):e00044-25. doi: 10.1128/spectrum.00044-25 (PMC12671204; doi:10.1128/spectrum.00044-25)
Supplement: Supplemental figures — Figure S1 to S4. [file spectrum.00044-25-s0001.docx]

**Figures**

**
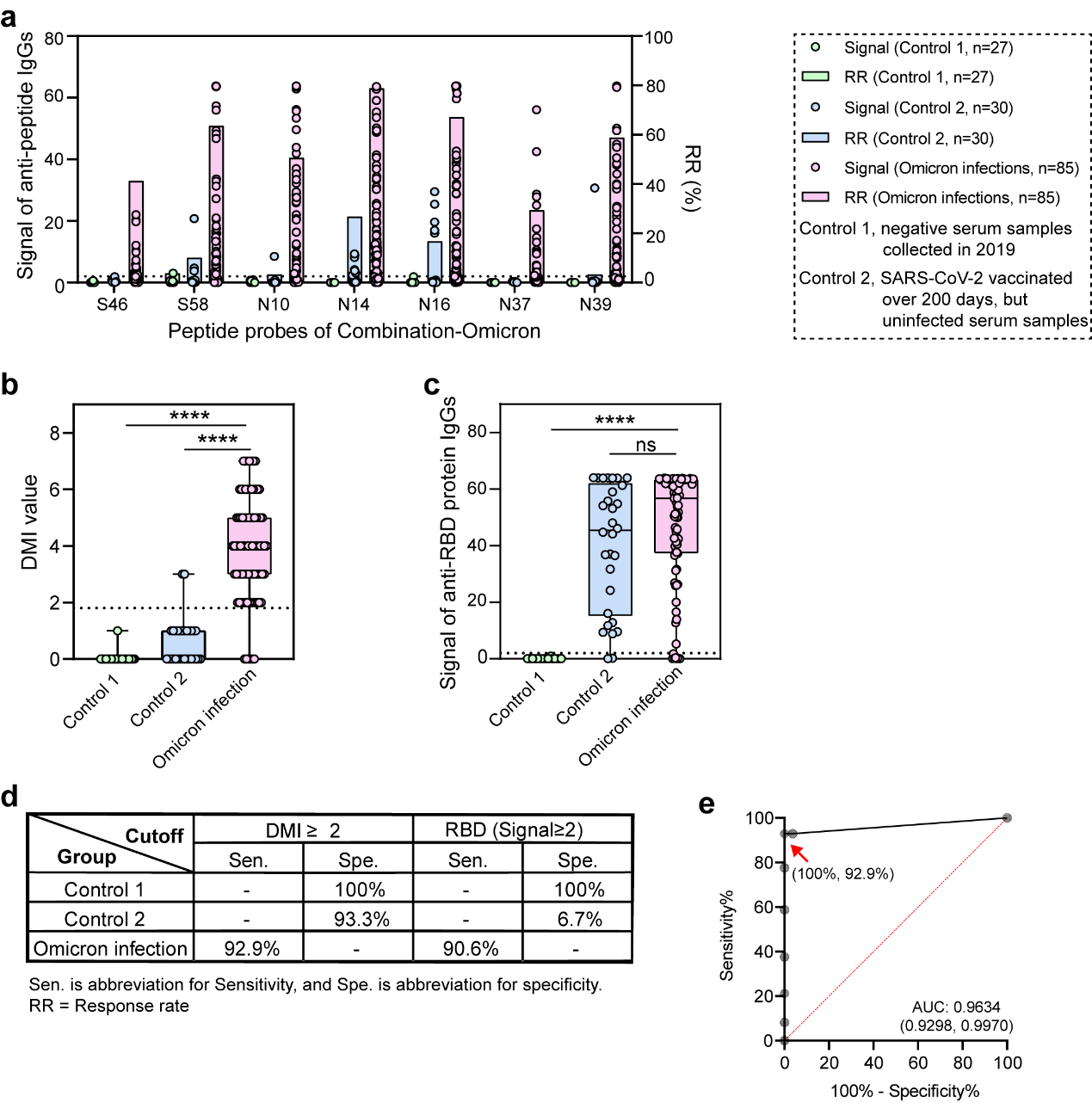
**

**Figure S1. The Combination-Omicron effectively differentiates between serum samples from individuals infected with the Omicron strain and those from uninfected or historically vaccinated individuals.** (a) The response signals and rates of the peptide probes (S46, S58, N10, N14, N16, N37, and N39) (*i.e.*, TPIs) in Combination-Delta were evaluated using serum samples from Control 1, Control 2, and Omicron-infected groups. (b) The DMI values were analyzed across the same groups. Significant differences in DMI values were observed between Control 1 and Omicron-infected serum samples, as well as between Control 2 and Omicron-infected groups (*p* < 0.001). (c) The response signals of the RBD protein (*i.e.*, PPIs) were evaluated using serum samples from the same groups. Significant differences in RBD response signals were observed between Control 1 and Omicron-infected groups (*p* < 0.001), with no significant differences between Control 2 and Omicron-infected groups. (d) When applying a DMI cutoff of ≥ 2 for anti-peptide antibodies or a signal value ≥ 2 for anti-protein antibodies, the groups exhibited varying specificities and sensitivities. At a DMI cutoff of ≥ 2, the specificities for the two control groups were 100% and 93.3%, respectively, and the sensitivity for the Omicron-infected group was 92.9%. Using RBD protein as a probe with a signal value ≥ 2, the two control groups had specificities of 100% and 6.7%, respectively, and the sensitivity for the Omicron-infected group was 90.6%. (e) Using a DMI cutoff ≥ 2, the Combination-Omicron demonstrated a specificity of 100% and a sensitivity of 92.9% for Control 1 and Omicron-infected samples, respectively.

**
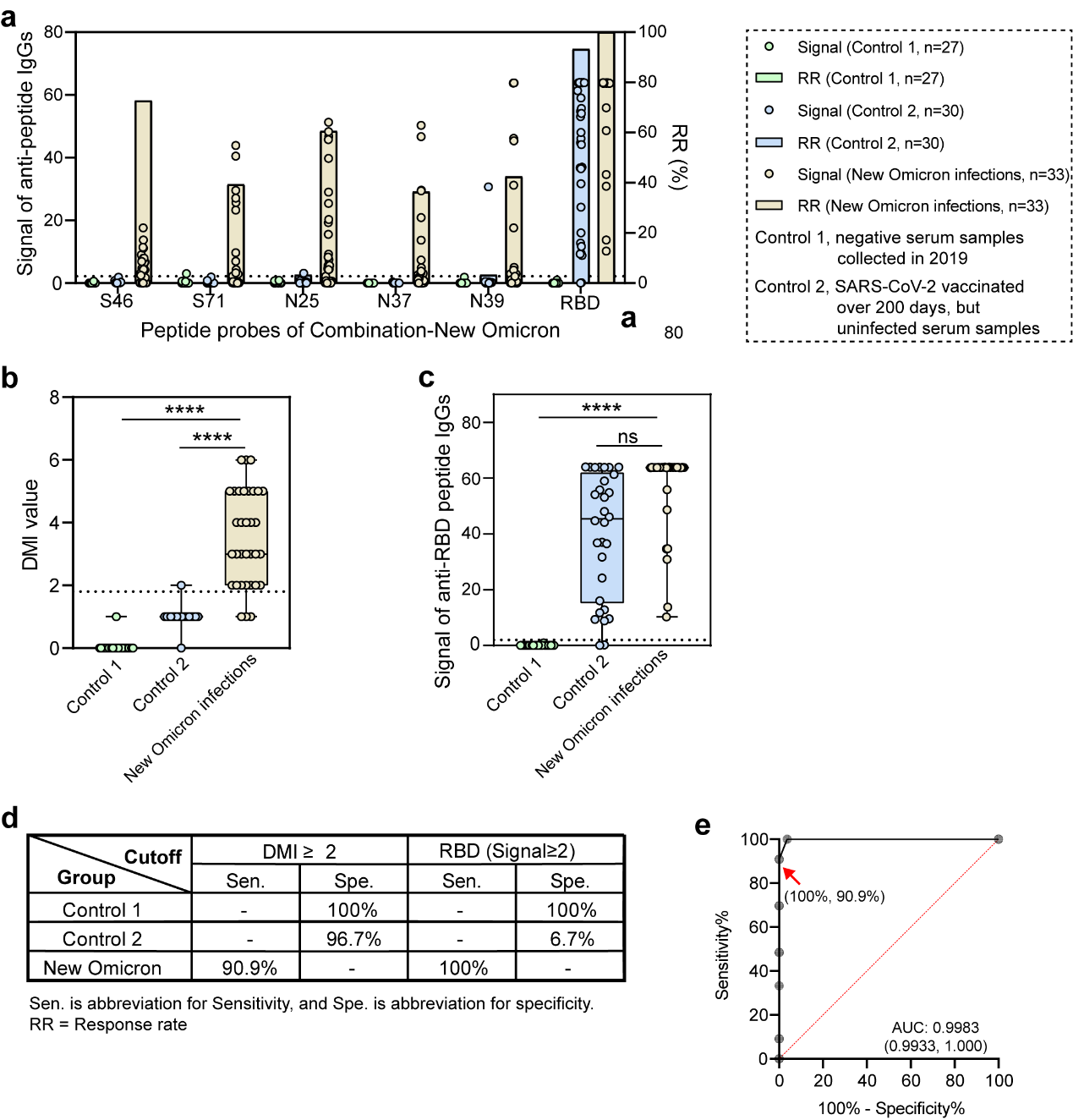
**

**Figure S2. The Combination-New Omicron effectively differentiates between serum samples from individuals infected with the New Omicron strain and those from uninfected or historically vaccinated individuals.** (a) The response signals and rates of the peptide probes (S46, S71, N25, N37, and N39) (*i.e.*, TPIs) in Combination-New Omicron were evaluated using serum samples from Control 1, Control 2, and New Omicron-infected groups. (b) The DMI values were analyzed across the same groups. Significant differences in DMI values were observed between Control 1 and New Omicron-infected serum samples, as well as between Control 2 and New-Omicron-infected groups (*p* < 0.001). (c) The response signals of the RBD protein (*i.e.*, TPIs) and were evaluated using serum samples from the same groups. Significant differences in RBD response signals were observed between Control 1 and New Omicron-infected groups (*p* < 0.001), with no significant differences between Control 2 and New Omicron-infected groups. (d) When applying a DMI cutoff of ≥ 2 for anti-peptide antibodies or a signal value ≥ 2 for anti-protein antibodies, the groups exhibited varying specificities and sensitivities. At a DMI cutoff of ≥ 2, the specificities for the two control groups were 100% and 96.7%, respectively, and the sensitivity for the New Omicron-infected group was 90.9%. Using RBD protein as a probe with a signal value ≥ 2, the specificities for the two control groups were 100% and 6.7%, respectively, and the sensitivity for the New Omicron-infected group was 100%. (e) Using a DMI cutoff ≥ 2, the Combination-New Omicron demonstrated a specificity of 100% and a sensitivity of 90.9% for Control 1 and New Omicron-infected samples, respectively.


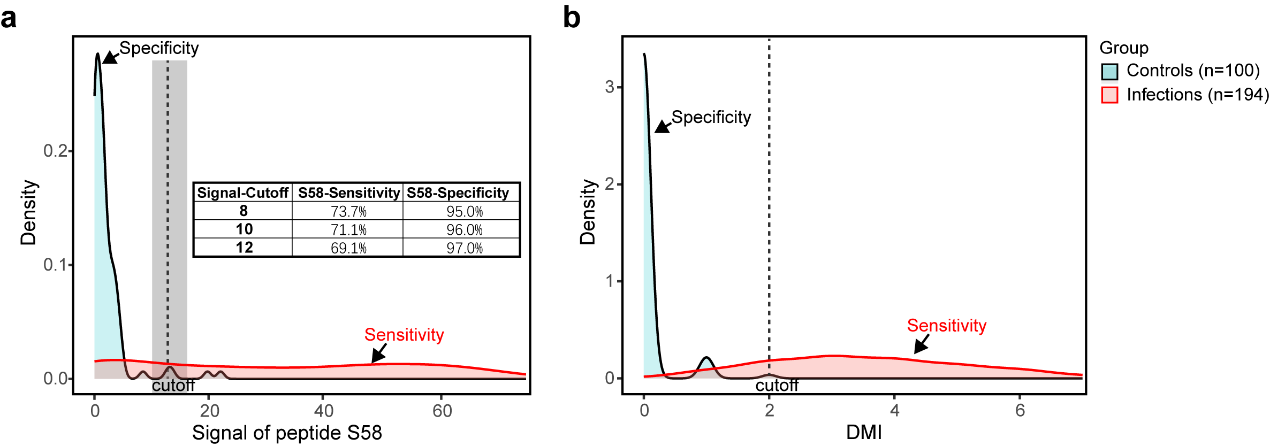


**Figure S3. DMI can simultaneously improve sensitivity and specificity compared to single peptide probe. (a)** Sensitivity and specificity of peptide S58 can be affected by cutoff. Here, when using signal cutoff = 10, peptide S58 showed a specificity of 96.0% and a sensitivity of 71.1% for 100 negative controls and 194 infectious serum samples, respectively. (b) DMI can simultaneously improve sensitivity and specificity. When using DMI cutoff = 2, the DMI showed a specificity of 99.0% and a sensitivity of 90.2% for 100 negative controls and 194 infectious serum samples, respectively.

**
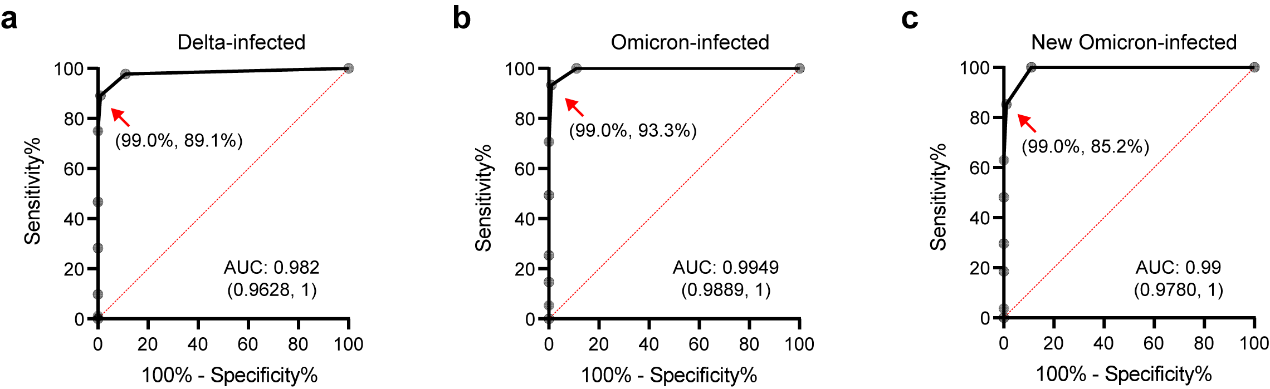
Figure S4. Detection Sensitivity and Specificity of PPHM_SARS-CoV-2_ Across Different SARS-CoV-2 Strains.** Using a cutoff of DMI ≥ 2, the PPHM_SARS-CoV-2_ assay’s sensitivity was: (a) 89.1% for Delta-infected serum samples, (b) 93.3% for Omicron-infected serum samples, and (c) 85.2% for New Omicron-infected serum samples, with a specificity of 99.0% for negative serum samples.
